# 3D-QSAR Design of New Escitalopram Derivatives for the Treatment of Major Depressive Disorders

**DOI:** 10.3797/scipharm.0912-22

**Published:** 2010-05-05

**Authors:** Speranta Avram, Catalin Buiu, Daniel M. Duda-Seiman, Corina Duda-Seiman, Dan Mihailescu

**Affiliations:** 1 Department of Anatomy, Animal Physiology and Biophysics, Faculty of Biology, University of Bucharest, 91-95, Splaiul Independentei, RO-76201, Bucharest, Romania; 2 Laboratory of Bioinformatics, Faculty of Control and Computers, Politehnica University of Bucharest, 313 Spl. Independentei, RO-060042, Bucharest, Romania; 3 Department of Medical Ambulatory, University of Medicine and Pharmacy “Victor Babes”, 49, B-dul C.D. Loga, RO-300020, Timisoara, Romania; 4 Department of Chemistry, Faculty of Chemistry-Biology-Geography, West University of Timisoara, 16, Pestalozzi, RO-300115, Timisoara, Romania

**Keywords:** Depression, Antidepressants, Membrane ions, SERT

## Abstract

Antidepressants are psychiatric agents used for the treatment of different types of depression being at present amongst the most commonly prescribed drug, while their effectiveness and adverse effects are the subject of many studies and competing claims. Having studied five QSAR models predicting the biological activities of 18 antidepressants, already approved for clinical treatment, in interaction with the serotonin transporter (SERT), we attempted to establish the membrane ions’ contributions (sodium, potassium, chlorine and calcium) supplied by donor/acceptor hydrogen bond character and electrostatic field to the antidepressant activity. Significant cross-validated correlation q^2^ (0.5–0.6) and the fitted correlation r^2^ (0.7–0.82) coefficients were obtained indicating that the models can predict the antidepressant activity of compounds. Moreover, considering the contribution of membrane ions (sodium, potassium and calcium) and hydrogen bond donor character, we have proposed a library of 24 new escitalopram structures, some of them probably with significantly improved antidepressant activity in comparison with the parent compound.

## Introduction

The World Health Organization reported that depressive disorders, particularly unipolar, are one of the leading causes of total disability-adjusted life-years (DALYs) worldwide [1]. Major depressive disorders have a number of causes: endothelial dysfunction, genetic risk factors, genetically determined personality factors, adverse childhood experiences [2] and is characterized, generally, by pervasive low mood, anxiety, inhibition of the cognitive process, loss of interest in a person's usual activities and suicidal behaviors [3]. The cost-effectiveness of more new antidepressant agents is superior to that of tricyclic antidepressants or selective serotonin reuptake inhibitors [4, 5]. Some of antidepressant agents are as follows [6]: serotonin-reuptake inhibitors – SSRIs, selective norepinephrine-reuptake inhibitors – NRIs, nonselective norepinephrine-reuptake inhibitors, dual-action reuptake inhibitors, monoamine oxidase inhibitors – MAOIs and new agents with complex mechanism of action. Recently, the British Association for Psychopharmacology has recommended [7] to choose those antidepressants that are better tolerated and safer in overdose. Thus, the combination of a SSRI with a newer agent represents the first line-treatment choice. Beyond their effectiveness in medical management of depression, SSRIs are recommended also in other conditions, such as anxiety disorders, panic disorders and social phobia [8]. Escitalopram is the newest and most selective of SSRIs approved by the FDA for depression treatment [9, 10]. In an Austrian study, six months after treatment start, the rate of clinical remission was higher for escitalopram treated patients than for citalopram (53.7% vs. 48.7%) and the cost for successfully treated patients with severe depression was with 24.4% lower for escitalopram than for citalopram [11].

The lack of knowledge regarding the three-dimensional structures of the membrane receptors and the high costs of antidepressants synthesis [12] can be real obstacles for psycho-pharmaceutical studies. Under these conditions, the quantitative structure-activity relationship (QSAR) represents a suitable way to predict the biological activity of new antidepressants, in the presence of different biological membrane components. At the level of the central nervous system, the influence of membrane ions (sodium, potassium, chlorine and calcium) upon drug action is critical. In our intention to augment the information about antidepressants’ mechanisms of action, in this paper we will establish by using 3D-QSAR the membrane ions’ contributions (sodium, potassium, chlorine and calcium) supply by donor/acceptor hydrogen bond and electrostatic field to the antidepressant activity of escitalopram, along with other serotonin-reuptake inhibitors (zimelidine, fluoxetine, paroxetine, sertraline, fluvoxamine), selective norepinephrine-reuptake inhibitors (reboxetine and atomoxetine), nonselective norepinephrine-reuptake inhibitors (desipramine, nortryptiline), dual-action reuptake inhibitors (amitriptyline, imipramine, venlafaxine, milnacipran, duloxetine), new agents with complex mechanism of action like mirtazapine, nefazodone and trazodone at the SERT active site. In this study we have not chosen a large number of molecules in the training set but other important criteria like clinical indication of antidepressants mentioned above, large range of affinity of antidepressants to SERT and also, chemical structure diversity, were considered.

Also, in our attempt to obtain novel escitalopram derivatives with fewer side-effects and a higher affinity to the serotonin transporter, a number of 24 new escitalopram derivatives were modeled and their affinity to SERT were predicted in accordance with estimated 3D-QSAR models. So, we chose to add a hydrophobic group (e.g. ethyl, *i*-propyl, propyl or *t*-butyl) to phenyl and also to the amine tail of escitalopram considering that the escitalopram derivatives antidepressant activity could be improved if the induced hydrophobic effect ensures an easier passage of the antidepressants through the biological membrane.

At present, few SAR studies are rather confined to small data sets and are using both the classical quantitative structure-activity relationship (2D-QSAR) [13] and 3D-QSAR approaches [14–16], giving rise to enhanced knowledge about antidepressant drugs and their interactions with different membrane receptors.

## Results and Discussion

### 3D-QSAR SERT antagonism activity model assessment of the sodium, H_2_O, OH-phenyl and nitrogen amide ions contribution

Initially, in the 3D-QSAR-ALMOND model, the individual atom probes sodium, OH-phenyl, nitrogen amide and water were used to predict the antidepressant biological activities, with a very poor correlation between experimental and in theory calculated binding affinities of antidepressants. Due to its negligible impact on the model, the above mentioned correlograms were removed from the initial set of probes. Thus, we considered necessary to supplement the reaction conditions with the presence of sodium, water, OH-phenyl and nitrogen amide ions in different combinations. It was noticed a clear improvement of statistic coefficients when combination of atom probes sodium and phenyl-OH, respective sodium and nitrogen amide atoms were used (Table 1).

The observed and predicted biological activities of the SERT antagonists for the training and test sets and also the difference between them considering the presence of Na and OH-phenyl are presented below (Table 2).

**Fig. 1.**
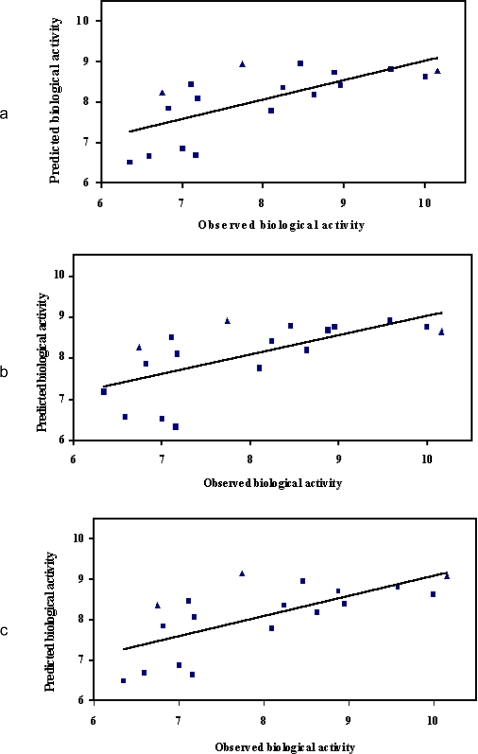
Correlation between observed and predicted binding affinities of antidepressants at the active SERT site. (a) Sodium and OH-phenyl atom probes. (b) Potassium and OH-phenyl atom probes. (c) Calcium and OH-phenyl atom probes (The molecules in the the test set are presented in triangles)

Data shown in Table 2 is supported by the correlations between experimental and calculated biological antidepressant activities when Na and OH -phenyl atom probes were considered (Figure.1a; the test set is presented in triangles). In this case, the best predictions of antidepressant biological activity were obtained for trazodone (residual value= −0.08) and fluoxetine (residual value=0.10) while PLS statistical results led to unsuitable correlations between predicted and observed biological activity for paroxetine (residual value = 1.38) and also for atomoxetine (residual value = −1.33). Also, the PLS regression resulted in a predictable satisfactory value of escitalopram SERT antagonism activity (residual value = 0.54).

### 3D-QSAR SERT antagonism activity model assessment of the potassium, H_2_O, OH-phenyl and nitrogen amide ions contribution

In a similar way with the previous 3D-QSAR models, a very poor correlation between observed and in predicted binding affinities of antidepressants were noticed when individual potassium, H_2_O and nitrogen amide atom probes were considered, but not when combination of potassium and OH-phenyl was used as grid atom probes (Table 1).

Analyzing data, it can be noticed that the presence of the potassium ion only is not enough to induce an efficient antidepressant effect. By consequence, when the reaction conditions were enriched adding simultaneously potassium and OH-phenyl, a clear improvement of statistic coefficients was noticed (r^2^ = 0.81, q^2^ = 0.56). The observed and predicted biological activities of the SERT antagonists for the training and test sets and also the difference between them (K-OH phenyl atom probes were considered) are presented in Table 2. Correlations between the two affinities types are illustrated in Figure 1b. Results indicated a high accuracy in the SERT antagonism prediction when antidepressants like trazodone (residual value= 0.01), fluoxetine (residual value= 0.17) and also escitalopram (residual value= 0.20) are studied. Similar with previous results, the predicted biological activity of paroxetine and also of atomoxetine in correlation with the observed biological activities were poor.

Chlorine is other important ion intervening in the electric signal transmission at the level of neuronal membrane. Thus, an objective of this study was to quantify the share of this ion in modulating the antidepressant effect of studied antidepressant molecules. As in the previous experiments, it was studied the effect of chlorine alone, or in association with other molecules (water, hydroxyl, amide nitrogen). PLS regression considering 3 latent variables (LV) resulted in a statistically unsatisfactory model (r^2^ less than 0.7, and q^2^ less than 0.50 respective) when (i) Cl-OH-phenyl, (ii) Cl-H_2_O and (iii) Cl-nitrogen amide were used in 3D-QSAR models. It was noticed that water, OH-phenyl and also nitrogen amide do not notably improve antidepressants’ activities. Due to its negligible impact on the model, 3D-QSAR models containing atom probes mentioned above have been removed from our study.

### 3D-QSAR SERT antagonism model used calcium, water, OH-phenyl, nitrogen amide as atom probes

In this study it was assessed the effect of a bivalent cation, calcium, in inducing the antidepressant activity. The reaction conditions were similar to the previous: it was assessed the effect of calcium with or without water, hydroxyl and amide nitrogen. The suitable q^2^ and r^2^ values are presented in Table 1. The observed and predicted biological activities of the SERT antagonists and the difference between them using calcium-OH phenyl atom probes is presented in Table 2 and also the correlation between experimental and theoretical binding affinities of antidepressants at the active SERT site, when calcium ion and hydroxyl group are used as atom probes are presented in Figure 1c.

The PLS regression led to a very good correlation between predicted and observed biological activities for fluoxetine (residual values = −0.1); trazodone (residual values= −0.1). In our intention to develop new escitalopram derivatives, we followed the correlation between predicted and observed biological actives of escitalopram. It is possible to notice that a residual value of 0.5 allowed us to predict the biological activity of escitalopram derivatives in respect with 3D-QSAR equation. The PLS statistic results proved that the presence of calcium and OH-phenyl have not improved the correlation between predicted and observed biological activity of paroxetine and atomoxetine.

### Novel structures of escitalopram derivatives with possible antidepressant profile

Nowadays, the major problem of antidepressants’ utilization is governed by severe side-effects which were already mentioned above, especially suicidal behavior and related thoughts induction, excepting the SSRI escitalopram. It has been discovered that in severe depressions, escitalopram has the advantage to have a more rapid action (depressive symptoms diminish after 1–2 weeks instead of 3–4 weeks) and less side effects compared to other SSRIs. Moreover, a meta-analysis of clinical trials database conducted by the escitalopram manufacturer Lundbeck found no indication that escitalopram would provoke suicidal behavior compared with placebo in patients with major depressive disorder and anxiety disorders, on the contrary, suicidal thoughts in the escitalopram group were significantly decreased. Due to aforementioned high importance of escitalopram, we have finally used a set of 24 potential new escitalopram derivatives and calculated their theoretical binding constants by using our above presented 3D-QSAR equations (Table 3).

The reliably predicted antidepressants’ activity at the SERT allows us to design new escitalopram derivatives which could be used as potential antidepressants. In our opinion an important improvement of the SERT antagonism activity should be obtained by facilitating the escitalopram membrane crossing as well as by generating more negative electrostatic contacts at the active site of the SERT. So, we enhanced the number of hydrophobic contacts of escitalopram, by adding allyl, ethyl, *i*-propyl, propyl, and *t*-butyl substituents as well as the number of hydrophilic contacts by adding halogen (F, Cl, Br), hydroxyl, nitro, methoxy or amide substituents.

The calculated values are suggesting a real improvement of escitalopram’s activity within the SERT for derivative 20 (R^1^=F, R^2^= ethyl, R^3^= ethyl, R^4^=H), derivative 21 (R^1^=F, R^2^= propyl, R^3^= propyl, R^4^=H) and derivative 23 (R^1^=F, R^2^= ethyl, R^3^= ethyl, R^4^=F) (Table 3).

There is no improvement for the escitalopram derivative 5 (R^1^= NH-CH=O, R^2^=CH_3_, R3= CH_3_, R4=H), derivative 16 (R1=F, R2=H, R3=H, R4=H) (Table 3) in interaction with SERT receptor. All these observations sustained the idea that the simultaneous presence of diethyl groups at the amine tail and difluoro atoms at the phenyl ring increased the antidepressant activity. On the other hand, it could be noticed that the drastically decrease of antidepressant activity is induced by the presence of dimethyl or dibutyl group linked by the amine tail or by the presence of primary amine. Same effect was recorded when the F, Cl- substituted phenyl or F, Br- substituted phenyl are considered.

## Experimental

### Data set

The binding constants of 18 antidepressant active compounds, with regard to serotonin transporter, were compiled from literature: (i) serotonin-reuptake inhibitors: zimelidine [17], fluoxetine [18], paroxetine [19], sertraline [18], fluvoxamine [18], escitalopram [18]; (ii) selective norepinephrine-reuptake inhibitors: reboxetine [20] and atomoxetine [20]; (iii) nonselective norepinephrine-reuptake inhibitors: desipramine [20], nortryptiline [17]; (iv) dual-action reuptake inhibitors: amitriptyline [21], imipramine [22], and the newer in the class – venlafaxine [21], milnacipran [21], duloxetine [21]; (v) new agents with complex mechanism of action like mirtazapine[23], nefazodone [24] and trazodone [22]. The list of studied compounds, and also their 2D – structures are shown in Table 4. Even if the number of compounds included in this study is not large, the data set was selected due to following criteria: (i) a large range of observed biological activities (6.35<pK_i_<10.15); (ii) favorable pharmacokinetic and pharmacodinamic properties covering the interactions with SERT compiled from literature [17–24]; (iii) the variety of substituents, covering as many as possible chemical classes of compounds.

### Molecular modeling and minimum energy of antidepressants

Three dimensional structures of studied compounds were obtained using the Sybyl 7 software. Further, in our study, energy minimized conformations of antidepressants were primarily established using the Maxim 2 minimization routine in Sybyl 7 [25] with Tripos force field, Conjugate-Gradient algorithm. After having obtained the appropriate conformations, the Gasteiger-Marsili partial charges of the compounds were loaded on the chemical structures from the Sybyl 7 dictionary.

### ALMOND strategy

These data were introduced into ALMOND [26] incorporated into Sybyl 7.3, where the calculation of the descriptors was performed. In our 3D-QSAR study default probes: sodium, potassium, chlorine and calcium (used as biological membrane ionic environment), water (used as electrostatic field), OH-phenyl (used as hydrogen bond donor) and nitrogen amide (used as hydrogen bond acceptor) were used in succession or in different combinations like (Na- H_2_O, Na-OH-phenyl, Na-N1 amide; K- H_2_O, K-OH-phenyl, K-N1 amide; Cl- H_2_O, Cl-OH-phenyl, Cl-N1 amide; Ca- H_2_O, Ca-OH-phenyl, Ca-N amide). Not all these combinations of atom probes were considered in the final models. Most of the Almond parameters were set to default values, for example, the grid spacing was equal to 0.5 A°, the smoothing window of the correlograms was set to 0.8, and the size of the correlograms was automatically established by the program.

### Chemometric analyses

The regression analysis was performed using the Partial Least Squares (PLS) [27, 28] algorithm within Sybyl 7. The optimum number of PLS components (latent variables, LV) was chosen by kept changes in the predictivity index (q^2^) model evaluated by applying the cross-validation procedure available in ALMOND. Also, SDEP (standard deviation of error prediction) [25] and SDEC (standard deviation of error calculation) [25] were evaluated. Further, the control criterions: r^2^ coefficient [25] was calculated in Sybyl ALMOND module by non-cross-validated method [27, 28].

### Training and testing sets

Interpretable models need to be constructed using a minimum set of compounds offering an enough large window of activities. For this reason we created two sets in which 15 molecules (6.35<pK_i_<10.15) were used as training set and 3 molecules were used for testing. The composition of both sets was kept unchanged during the study as follows: training set comprised amitriptiline, atomoxetine, escitalopram, fluoxetine, fluvoxamine, imipramine, milnacipram, mirtazepine, nefazodone, paroxetine, reboxetine, sertraline, trazodone, venlafaxine, zimelidine while desipramine, nortryptiline and duloxetine belonged to the test set.

### Modeling new escitalopram derivatives with potential affinity to SERT

Due to the before mentioned high importance of escitalopram as antidepressant agent, a set of 24 new escitalopram derivatives was created in order to predict their antagonism activity to SERT using our above presented 3D-QSAR models. In this respect, we followed two strategies: first, we generated more negative electrostatic contacts by adding halogen (F, Cl, Br), hydroxyl, nitro, methoxy or amide substituents at R1, respective R4 positions (see Table 3) of and, secondly, we enhanced the number of hydrophobic contacts of escitalopram, by adding allyl, ethyl, i-propyl, propyl, and t-butyl substituents at R^2^–R^4^ positions (see Table 3). The strategy of molecular modeling and minimum energy of escitalopram derivatives were performed in Sybyl 7.3 in the similar manner presented in 3.2.

## Conclusions

ALMOND-based 3D-QSAR models can give different kind of information like reliable prediction of the biological activity of compounds belonging to the data set and chemical interpretation of the obtained results. In this paper we have reported alignment-independent 3-D QSAR studies on a series of 18 antidepressants already accepted in the clinical treatment and also of new 24 escitalopram derivatives against SERT transporter into QSAR models. In our study, 3D-QSAR-ALMOND models were used to elucidate the most important physicochemical properties which are responsible for the binding properties of 18 antidepressant agents at SERT active site. An analysis of our QSAR results on antidepressants interactions with serotonin transporter (SERT) brings up a number of points of interest. In our study, seven atom probes (biological membrane ions (sodium, chlorine, potassium, and calcium), water (as electrostatic field), nitrogen amide (as hydrogen-bond donor) and OH-phenyl (used as hydrogen bond acceptor) were considered. The PLS significant statistic results were obtained when ions atom probes were considered in combination with OH –phenyl and N1 amide.

Comparing the PLS statistic results it was possible to put in evidence some similarities and differences between the models. The influences of these descriptors to binding affinities could be laid down in five QSAR equations which show that the binding affinity of an antidepressant can be influenced by the simultaneous presence of the cations and of the hydroxyl anion, while the simultaneous presence of water atoms and of the cations can influence the binding affinity, but certainly in a weaker manner than the presence of hydroxyl.

Our results indicate that the judicious modulation of the physicochemical properties, particularly hydrophobic and electronic ones, may be very useful in designing new antidepressant drugs. Considering the above set of 24 new potential escitalopram structures, the established equations could be possibly used as a guidance to enhance or diminish K_i_ values according to the particular biological need. In our opinion, these compounds represented a suggestion for further clinical and also, molecular simulation studies.

## Figures and Tables

**Tab. 1.** Summary of the ALMOND statistical parameters

**Statistical parameters**	**Sodium and OH-phenyl atom probes**	**Potassium and OH-phenyl atom probes**	**Calcium and OH-phenyl atom probes**	**Sodium and nitrogen amide atom probes**	**Calcium and nitrogen amide atom probes**
No. of molecules in training set	15	15	15	15	15
q^2^	0.60	0.56	0.60	0.56	0.57
r^2^	0.80	0.81	0.80	0.82	0.82
SDEP	0.69	0.72	0.70	0.72	0.72
SDEC	0.47	0.46	0.48	0.45	0.46

q^2^ … cross-validated correlation coefficient; r^2^-fitted correlation coefficient; SDEP- standard deviation of error prediction; SDEC- standard deviation of error calculation.

**Tab. 2.** Observed and predicted affinities of antidepressants at SERT active site and difference between them (residual value)

**Antidepressant**	**Observed affinities at SERT active site**	**Predicted affinities at SERT active site**			**Difference between observed and predicted affinities**		

		**Training set**					

		**A**	**B**	**C**	**A**	**B**	**C**
amitriptiline	8.46	8.94	8.77	8.95	−0.48	−0.31	−0.49
atomoxetine	7.11	8.44	8.51	8.45	−1.33	−1.40	−1.34
escitalopram	8.95	8.41	8.75	8.39	0.54	0.20	0.56
fluoxetine	8.24	8.34	8.41	8.34	−0.10	−0.17	−0.10
fluvoxamine	8.63	8.18	8.21	8.17	0.45	0.42	0.46
imipramine	8.88	8.73	8.69	8.71	0.15	0.19	0.17
milnacipram	6.82	7.84	7.86	7.84	−1.02	−1.04	−1.02
mirtazepine	7.00	6.84	6.53	6.86	0.16	0.47	0.14
nefazodone	7.16	6.68	6.33	6.64	0.48	0.83	0.52
paroxetine	10.00	8.62	8.76	8.61	1.38	1.24	1.39
reboxetine	6.35	6.51	7.18	6.48	−0.16	−0.83	−0.13
sertraline	9.58	8.81	8.94	8.81	0.77	0.64	0.77
trazodone	6.59	6.67	6.58	6.69	−0.08	0.01	−0.10
venlafaxine	8.10	7.78	7.78	7.77	0.32	0.32	0.33
zimelidine	7.18	8.08	8.11	8.07	−0.90	−0.93	−0.89
		
		**Test set**					
		
desipramine	10.15	8.76	8.66	9.09	1.39	1.49	1.06
nortryptiline	7.74	8.95	8.92	9.15	−1.21	−1.18	−1.41
duloxetine	6.74	8.23	8.28	8.37	−1.49	−1.54	−1.63

A sodium and OH-phenyl atom probes were used;

B potassium and OH-phenyl atom probes were used;

C calcium and OH-phenyl atom probes were used.

**Tab. 3.** Predicted affinity of new escitalopram derivatives to SERT active site. The residual values (the biological activity for novel escitalopram derivatives differences to the parent biological activity) are in brackets.
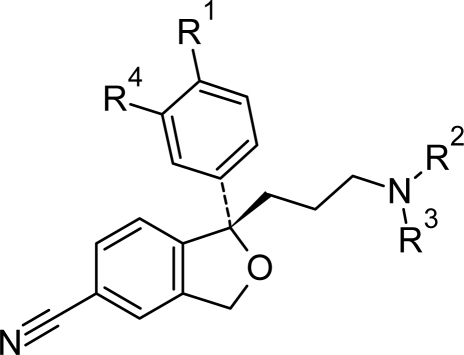

**Escitalopram derivative**	**Substituents**				**pK_i_^a^**	**pK_i_^b^**	**pK_i_^c^**

	**R^1^**	**R^2^**	**R^3^**	**R^4^**			
Derivative 1	Cl	CH_3_	CH_3_	H	9.05(0.1)	9.02(0.07)	9.03(0.08)
Derivative 2	Br	CH_3_	CH_3_	H	9.04(0.09)	9.01(0.06)	9.03(0.08)
Derivative 3	OH	CH_3_	CH_3_	H	8.95(0)	8.96(0.01)	8.94(−0.01)
Derivative 4	CH_3_	CH_3_	CH_3_	H	8.99(0.04)	8.98(0.03)	8.97(0.02)
Derivative 5	NH-CH=O	CH_3_	CH_3_	H	8.72(−0.23)	8.76(−0.19)	8.71(−0.24)
Derivative 6	NO2	CH_3_	CH_3_	H	9.05(0.1)	9.04(0.09)	9.04(0.09)
Derivative 7	OCH_3_	CH_3_	CH_3_	H	9.02(0.07)	9.06(0.11)	9.01(0.06)
Derivative 8	F	CH_3_	CH_3_	F	9.09(0.14)	9.18(0.23)	9.08(0.13)
Derivative 9	F	CH_3_	CH_3_	Cl	9.08(0.13)	9.14(0.19)	9.07(0.12)
Derivative 10	F	CH_3_	CH_3_	allyl	8.94(−0.01)	8.95(0)	8.93(−0.02)
Derivative 11	F	CH_3_	CH_3_	ethyl	9.16(0.21)	9.16(0.21)	9.15(0.2)
Derivative 12	F	CH_3_	CH_3_	*i*-propyl	8.91(−0.04)	8.87(−0.08)	8.9(−0.05)
Derivative 13	F	CH_3_	CH_3_	OCH3	9.12(0.17)	9.17(0.22)	9.11(0.16)
Derivative 14	F	CH_3_	CH_3_	*t*-butyl	9.06(0.11)	9.04(0.09)	9.05(0.1)
Derivative 15	F	CH_3_	CH_3_	OH	8.95(0)	9.03(0.08)	8.94(−0.01)
Derivative 16	F	H	H	H	8.75(−0.2)	8.68(−0.27)	8.73(−0.22)
Derivative 17	F	H	CH_3_	H	8.99(0.04)	8.94(−0.01)	8.98(0.03)
Derivative 18	F	allyl	CH_3_	H	8.89(−0.06)	8.86(−0.09)	8.88(−0.07)
Derivative 19	F	*i*-propyl	CH_3_	H	8.98(0.03)	8.94(−0.01)	8.96(0.01)
Derivative 20	F	ethyl	ethyl	H	9.38(0.43)	9.35(0.4)	9.37(0.42)
Derivative 21	F	propyl	propyl	H	9.36(0.41)	9.28(0.33)	9.34(0.39)
Derivative 22	F	*t*-butyl	*t*-butyl	H	9.23(0.28)	9.14(0.19)	9.22(0.27)
Derivative23	F	ethyl	ethyl	F	9.43(0.48)	9.45(0.5)	9.41(0.46)
Derivative24	F	*t*-butyl	*t*-butyl	F	9.32(0.37)	9.31(0.36)	9.31(0.36)

a Na-OH-phenyl atom probes;

b K-OH-phenyl atom probes;

c Ca-OH-phenyl atom probes.

**Tab. 4.** The chemical structures and observed biological activity of antidepressant agents at the SERT site pK_iSERT_

**Antidepressant**	**2D-STRUCTURE**	**pK_i SERT_**

**serotonin-reuptake inhibitors**		
zimelidine	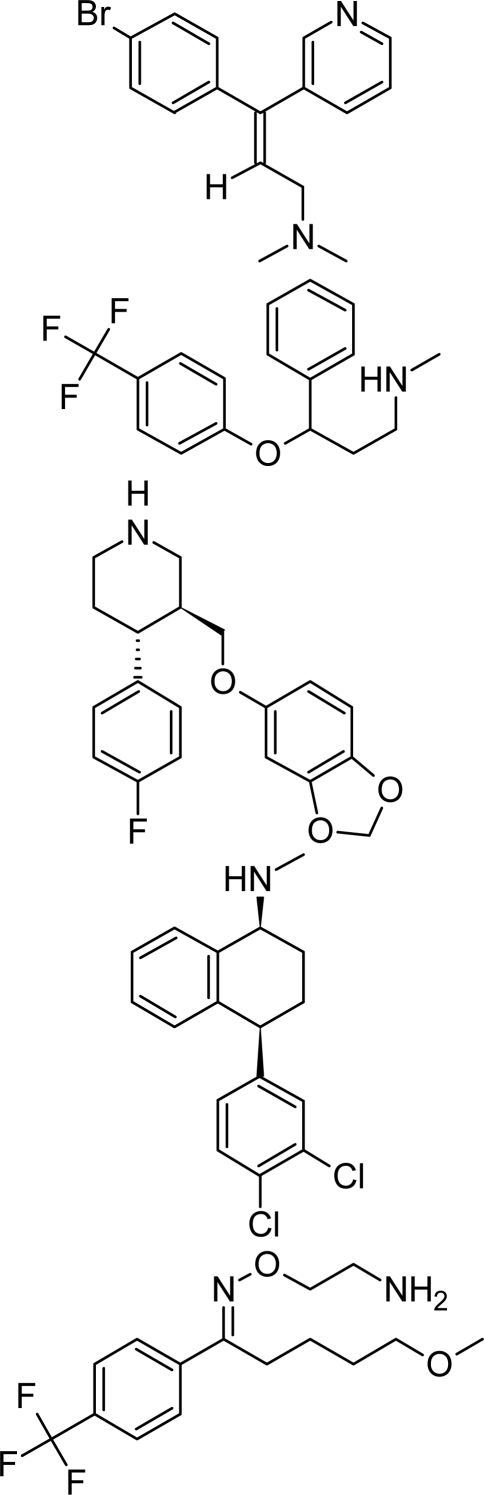	7.18 [17]
fluoxetine		8.24 [18]
paroxetine		10 [19]
sertraline		9.58 [18]
fluvoxamine		8.63 [18]
escitalopram	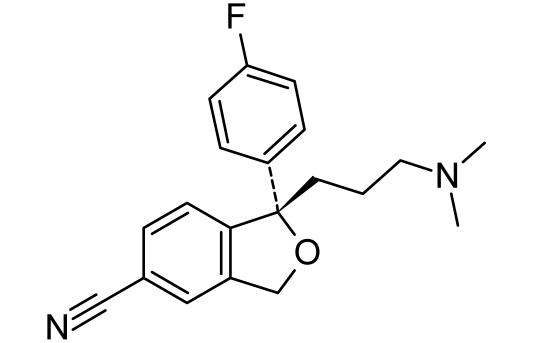	8.95 [18]

**selective norepinephrine-reuptake inhibitors**		

reboxetine	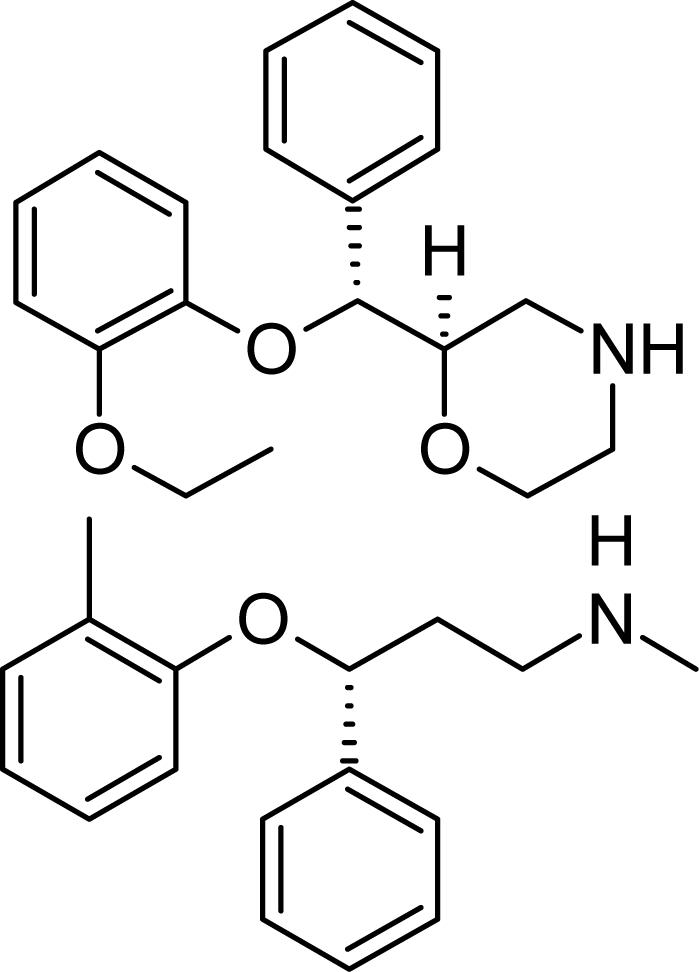	6.35 [20]
atomoxetine		7.11 [20]

**nonselective norepinephrine-reuptake inhibitors**		

desipramine	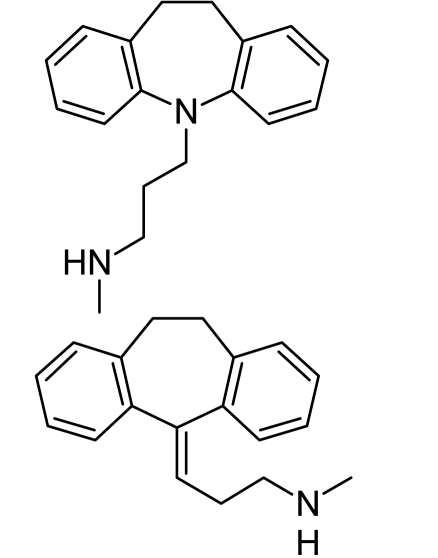	6.74 [20]
nortryptiline		7.74 [17]

**dual-action reuptake inhibitors**		

amitriptyline	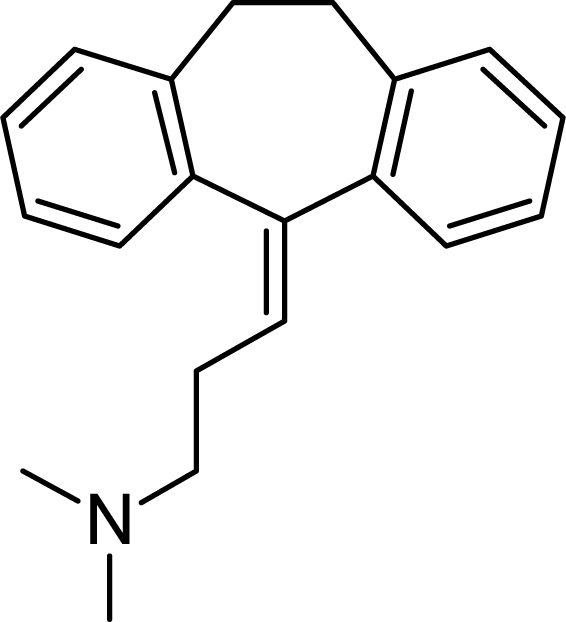	8.46 [21]
imipramine	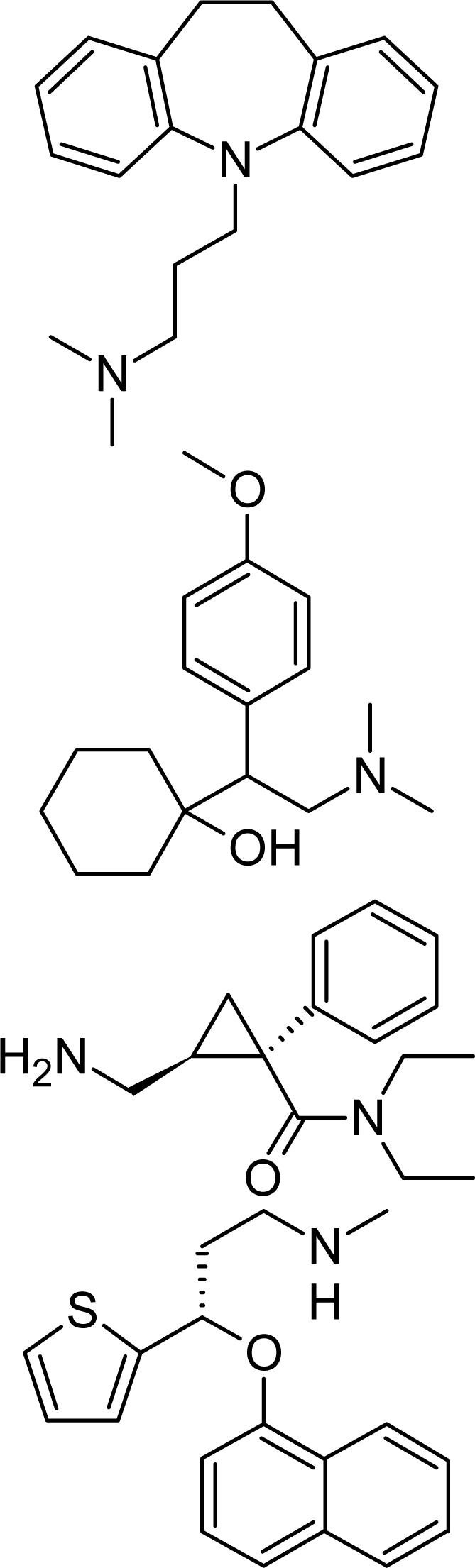	8.88 [22]
venlafaxine		8.10 [21]
milnacipran		8.07 [21]
duloxetine		10.15 [21]

**new agents with complex mechanism of action**		

mirtazapine	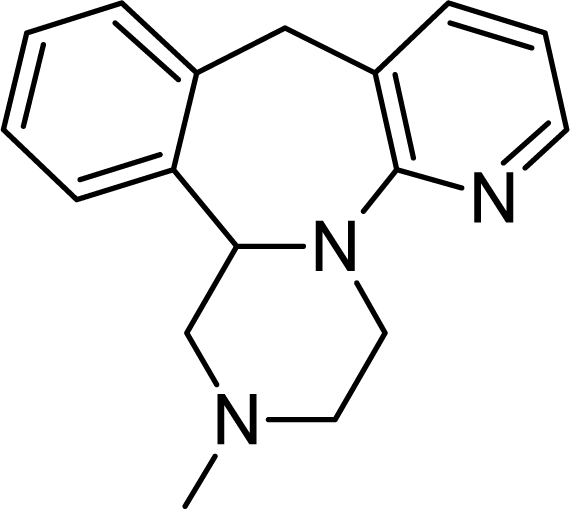	7.00 [23]
nefazodone	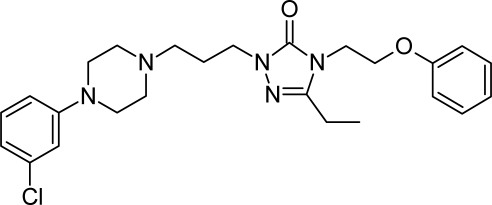	7.16 [24]
trazodone	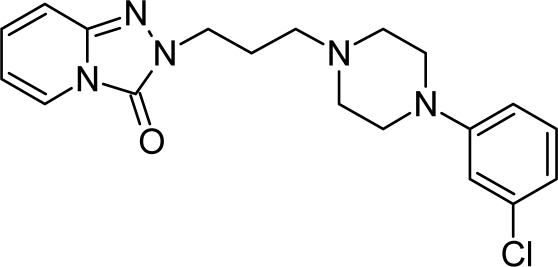	6.59 [22]

The references used for observed biological activity of antidepressants are presented in brackets.
